# Negative anti-phospholipase A2 receptor antibody status at three months predicts remission in primary membranous nephropathy

**DOI:** 10.1080/0886022X.2022.2033265

**Published:** 2022-02-16

**Authors:** Gabriel Stefan, Simona Stancu, Adrian Zugravu, Otilia Popa, Dalia Zubidat, Nicoleta Petre, Gabriel Mircescu

**Affiliations:** a University of Medicine and Pharmacy Carol Davila, Bucharest, Romania; b Dr. Carol Davila Teaching Hospital of Nephrology, Bucharest, Romania

**Keywords:** Anti-phospholipase A2 receptor antibody, primary membranous nephropathy, three months negativization of anti-phospholipase A2 receptor antibody, kidney survival

## Abstract

**Background:**

The value of anti-phospholipase A2 receptor antibody (anti-PLA2R ab) monitoring at 3 months after diagnosis in membranous nephropathy (MN) remains uncertain.

**Methods:**

We retrospectively examined the outcome on 1 August 2020 of 59 adult patients (age 54 (44, 68) years, 69% male, SCr 1.0 (0.9, 1.3) mg/dL) diagnosed with MN (kidney biopsy, positive serum anti-PLA2R ab). The outcomes were: kidney survival; partial and/or complete remission.

**Results:**

Most of the studied patients (97%) received immunosuppression, cyclophosphamide regimens were the most frequent (87%), followed by cyclosporine (10%). The median time to remission was 12.0 months and the cumulative remission rates were 34% at 6, 54% at 12, and 73% at 24 months. Forty (69%) patients had negative anti-PLA2R ab at 3 months, they had similar age, serum creatinine, albumin, proteinuria, and treatment with the group with positive ab at 3 months. In the Cox proportional hazard model, three months anti-PLA2R ab negativization (HR 0.4 (95%CI 0.1, 0.9)) was an independent predictor for remission, while baseline hypoalbuminemia (HR 3.0 (95%CI 1.5, 5.7)) was associated with absence of remission. Six (10%) patients died, mostly due to cardiovascular disease and infections. A total of five (9%) patients started dialysis. Mean kidney survival time was 50.3 months and there was no survival difference in relation to baseline anti-PLA2R ab titer (*p* .09) or 3 months negativization (*p* .8).

**Conclusions:**

Three months anti-PLA2R ab negativization seems to be a late predictor of remission, and lower serum albumin at diagnosis is an early marker for remission absence. **Abbreviations:** anti-P LA2R ab, anti-phospholipase A2 receptor antibody; eGFR, estimated glomerular filtration rate; ESKD, end stage kidney disease; MN, membranous nephropathy; NELL-1, neural epidermal growth factor-like 1 protein; RAAS: renin–angiotensin–aldosterone system; RBC: red blood cells; RRT, renal replacement therapy; T HSD7A, thrombospondin type-1 domain containing 7A

## Introduction

Primary membranous nephropathy (MN) is a kidney-specific autoimmune disease associated with autoantibodies against certain podocyte membrane antigens, resulting in immune complex deposits on the outer face of glomerular basement membrane, consequent damage of the glomerular filtration barrier and nephrotic syndrome [1,2].

The most frequent antigen found on the podocyte membrane in MN is the M-type phospholipase A2 receptor (PLA2R) [1]. Anti-phospholipase A2 receptor (anti-PLA2R) antibodies testing revolutionized the management of MN in the past 10 years on all aspects, from diagnosis to prognosis and treatment [2–6]. However, the additive value of anti-PLA2R antibodies titer monitoring during the first three-to-six-month after diagnosis remains uncertain, and there are insufficient data to guide the use of antibodies levels in making initial treatment decisions [1].

Therefore, we aimed to evaluate the predictive value for remission, renal, and patient survival of anti-PLA2R antibodies titer at baseline and at three-months after diagnosis in primary MN.

## Methods

### Patients and study design

This retrospective unicentric study included consecutive patients newly diagnosed with MN by kidney biopsy and at least one serum anti-PLA2R antibody test at diagnosis between 1 January 2016 and 31 December 2019.

**Figure 1.**
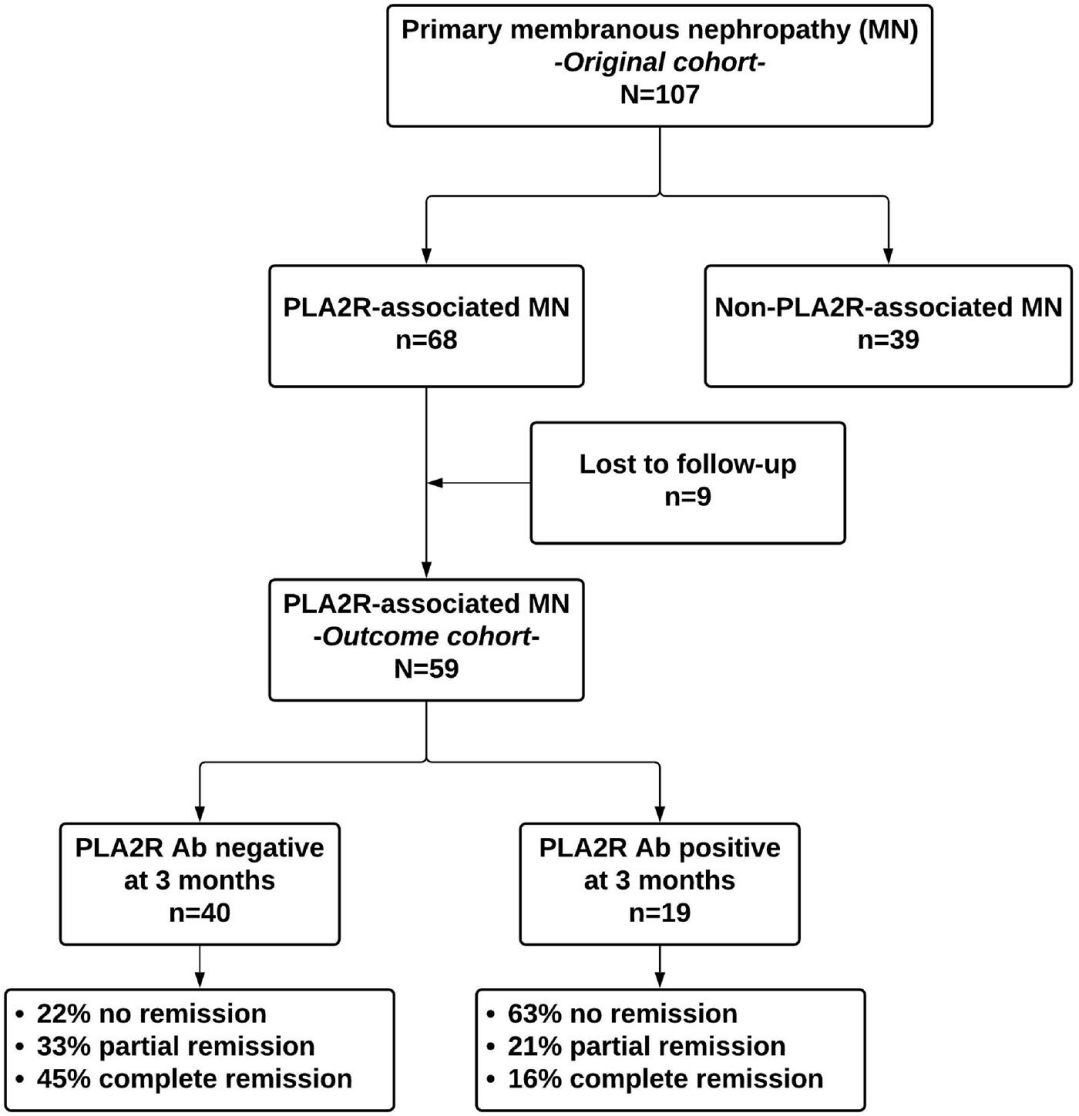
Study diagram. Ab: antibodies; PLA2R: phospholipase A2 receptor; MN: membranous nephropathy.

The patients were followed from kidney biopsy to renal replacement therapy (RRT) initiation, death, loss to follow up or until 1 August 2020. For the outcome analysis, only patients with first episode MN, and who were followed for at least 6 months were included (Figure 1).

Extensive assessment of each case excluded secondary causes of MN, such as tumors, systemic diseases, viral infections, and previous medication.

The primary endpoint was the composite of complete or partial remission. Complete remission was defined as proteinuria under 0.5 g per 24 h, serum albumin of at least 3.5 g per deciliter and stable eGFR. Partial remission was defined as a reduction in proteinuria of at least 50% from baseline with proteinuria between 0.5 g and 3.5 g per 24 h and stable eGFR [1]. A stable GFR was defined as an eGFR remaining unchanged or declines by <15% during follow-up.

Relapse was defined as the reappearance of a more than 3.5 g per 24 h proteinuria after a complete or a partial remission [1].

Electronic medical records were reviewed for demographics, presentation characteristics, thrombotic complications, outcome data, and laboratory parameters (i.e., serum creatinine, proteinuria, serum albumin, blood lipids, and inflammation).

The indication and choice of immunosuppressive treatment was at discretion of the nephrologist in charge of the patient. Since all the patients were included in the study at diagnosis, there were no patients that received immunosuppressive therapy before study inception.

### Anti-PLA2R antibodies assessment

Serum levels of total anti-PLA2R IgG antibodies were measured by ELISA, using a test developed by EUROIMUN AG (Lübeck, Germany). According to manufacturer, the test is positive at a level >14 RU/mL. Accordingly, patients were classified at diagnosis as being negative or positive for anti-PLA2R antibodies based on this cutoff (Figure 1). Also, negativization at three months was defined as a serum anti-PLA2R titer lower than 14 RU/mL [7].

### Kidney biopsy study

For each biopsy specimen, light microscopy, immunofluorescence, and electron microscopy were routinely performed.

The histological analysis included an in-depth review of the glomerular, tubulointerstitial, and vascular compartments. Definitions of histologic variables used in our study were derived from the Mayo Clinic/Renal Pathology Society Consensus [8] and are depicted in S1 Table S1.

Also, we calculated the kidney biopsy chronicity score as proposed by Sethi et al. which standardized a semiquantitative grading for glomerulosclerosis (0–3), interstitial fibrosis (0–3), tubular atrophy (0–3), and arteriolosclerosis (0/1). Total chronicity score is the sum of individual chronicity scores of each renal compartment [9].

### Statistical analysis

Descriptive statistics were summarized as mean ± SD or median (quartile 1, quartile 4) for continuous variables, and frequency distribution is presented as percentages for categorical variables. Group comparisons were performed with Student’s *t*-test, *χ*^2^ test, and Mann–Whitney’s *U*-test, as appropriate.

Survival analyses were conducted with the Kaplan–Meier method, and the log rank test was used for comparison. Univariate and multivariate Cox proportional hazard analyses were performed to identify independent predictors of the primary and secondary endpoints. If the *p* value of the candidate predictor in univariate survival analysis was <.05, this predictor was included in multivariable Cox regression model. Results were expressed as hazard ratio (HR) and 95% confidence interval (CI). Moreover, we used two methods in order to test for collinearity among our predictor variables: (i) the variance inflation factor (VIF), where VIF <10 is desirable; (ii) the absolute value of correlation coefficients, where |*r*| or |*rs*| <0.7 is desirable. There was no significant collinearity between the variables used in Cox proportional hazard models.

All statistical tests were two sided, and a *p* < .05 was considered significant. Statistical analyses were performed using the SPSS program (SPSS version 26, Chicago, IL).

### Ethical code

The study was conducted with the provisions of the Declaration of Helsinki and the protocol was approved by the local ethics committee (Dr Carol Davila Teaching Hospital of Nephrology, Bucharest, Romania, approval number 2021-010). Since all data were anonymized, informed consent was not obtained from individual patients.

## Results

### Study population

One hundred and seven patients were identified as having primary MN at kidney biopsy and were tested for anti-PLA2R antibodies (i.e., *original cohort*). Sixty-eight (64%) tested positive for anti-PLA2R antibodies. However, only 59 (55%) patients were followed for at least six months and were included in the outcome analysis (i.e., *outcome cohort*) (Figure 1).

### Original cohort

The baseline characteristics of the *original cohort* are presented in S1 Table S2. The median age was 56 years at time of kidney biopsy and there was a male predominance (65%).

Arterial hypertension was present in 60% of the patients and only 10% had diabetes mellitus. Most of the patients (94%) had full blown nephrotic syndrome at diagnosis. However, thrombotic complications were present only in 12% of the studied patients and included: pulmonary embolism, coronary artery thrombosis, and deep vein thrombosis.

Two-thirds of the patients with MN (64%) were positive for PLA2R antibodies. Proteinuria, serum albumin, and kidney function were similar regardless of the anti-PLA2R antibodies cutoffs used (<2, 2–14, >14 RU/mL) (S1 Figure S1).

There were no differences between positive and negative anti-PLA2R antibodies groups regarding age, sex, comorbidities, kidney function, nephrotic syndrome severity, and outcome (i.e., death and RRT initiation). However, the anti-PLA2R antibodies negative patients were less frequently treated with immunosuppressants (S1 Table S2).

### Outcome cohort

The baseline characteristics of patients from the outcome cohort are depicted in Table 1. The median age at kidney biopsy was 54 years, 69% were male and the median follow-up time was 21.0 (95%CI, 14.2–27.7) months. More than half had hypertension and only 9% diabetes mellitus. The median serum creatinine was 1 mg/dL and all patients had nephrotic syndrome. The anti-PLA2R antibodies titer median at diagnosis was 185 [62–485] RU/mL.

**Table 1.** Outcome cohort patients’ characteristics at baseline, according to PLA2R antibodies negativization at three months.

	All (*N* = 59)	Three months anti-PLA2R ab negativization		
		Yes (*n* = 40)	No (*n* = 19)	*p*
Age (years)	54 [44–68]	57 [48–68]	53 [43–67]	.3
Male sex (%)	69	63	79	.4
Hypertension (%)	58	58	58	.9
Diabetes mellitus (%)	9	5	16	.1
Charlson score	0 [0–2]	0 [0–2]	0 [0–1]	.9
Thrombotic complications (%)	12	15	5	.2
Anti-PLA2R Ab (RU/mL)	185 [62–485]	103.5 [53.8–270.9]	485.2 [256–1031]	.004
Serum creatinine (mg/dL)	1.0 [0.9–1.3]	1.0 [0.8–1.3]	1.0 [0.9–1.3]	.9
Serum albumin (g/dL)	2.4 [2.0–2.8]	2.5 [2.0–2.9]	2.3 [2.0–2.6]	.6
Proteinuria (g/g)	7.2 [4.9–12.6]	7.2 [4.2–13.0]	6.8 [5.5–10.3]	.6
Hematuria (RBC/mm^3^)	12 [5–55]	11 [5–35]	20 [5–65]	.2
C-reactive protein (mg/L)	1 [1–5]	1 [1–5]	2 [1–11]	.3
Cholesterol (mg/dL)	348 [281–463]	336 [275–451]	382 [304–517]	.5
Triglycerides (mg/dL)	223 [168–282]	207 [163–272]	239 [173–318]	.2
*Kidney biopsy*				
MN stage (%)				.4
I	3	3	5	
II	59	60	58	
III	31	27	37	
IV	7	10	0	
Total chronicity score	3 [1–5]	3 [1–4]	2 [1–8]	.9
*Treatment*				
Immunosuppression (%)				.3
Absent	3	5	0	
Corticotherapy only	7	10	0	
Cyclophosphamide^a^	87	83	95	
Cyclosporine^b^	3	2	5	
RAAS blockade (%)	39	40	42	.3
*Outcome*				
Response to therapy (%)				.008
No remission	36	22	63	
Partial remission	36	33	21	
Complete remission	28	45	16	
Death (%)	10	10	11	.9
RRT initiation (%)	9	8	11	.6

MN: membranous nephropathy; PLA2R: phospholipase A2 receptor; RAAS: renin–angiotensin–aldosterone system; RBC: red blood cells; RRT: renal replacement therapy.

^a^ Cyclophosphamide (cyclical): methylprednisolone 1 g iv 3 consecutive days in months 1, 3, and 5, prednisone 0.5 mg/kg/d in months 1, 3, and 5; CFM 2.5 mg/kg/d in months 2, 4, and 6. Cyclophosphamide (intravenous pulse): CFM at a dose of 600 mg/m^2^ every 4 weeks – for up to 6 months; in conjunction with prednisolone at a dose of 0.75 mg/kg daily (up to 60 mg/day) – with gradual tapering to 0.5 mg/kg/day by 3 months and 0.1 mg/kg/day by 6 months.

^b^ Cyclosporine 3.5 mg/kg/d and prednisone 10 mg/d – 6–12 months.

Fifty-six percent of patients received RAAS blockade and almost all (97%) received immunosuppression.

### Anti-PLA2R antibodies titer and clinical presentation at diagnosis

**Figure 2.**
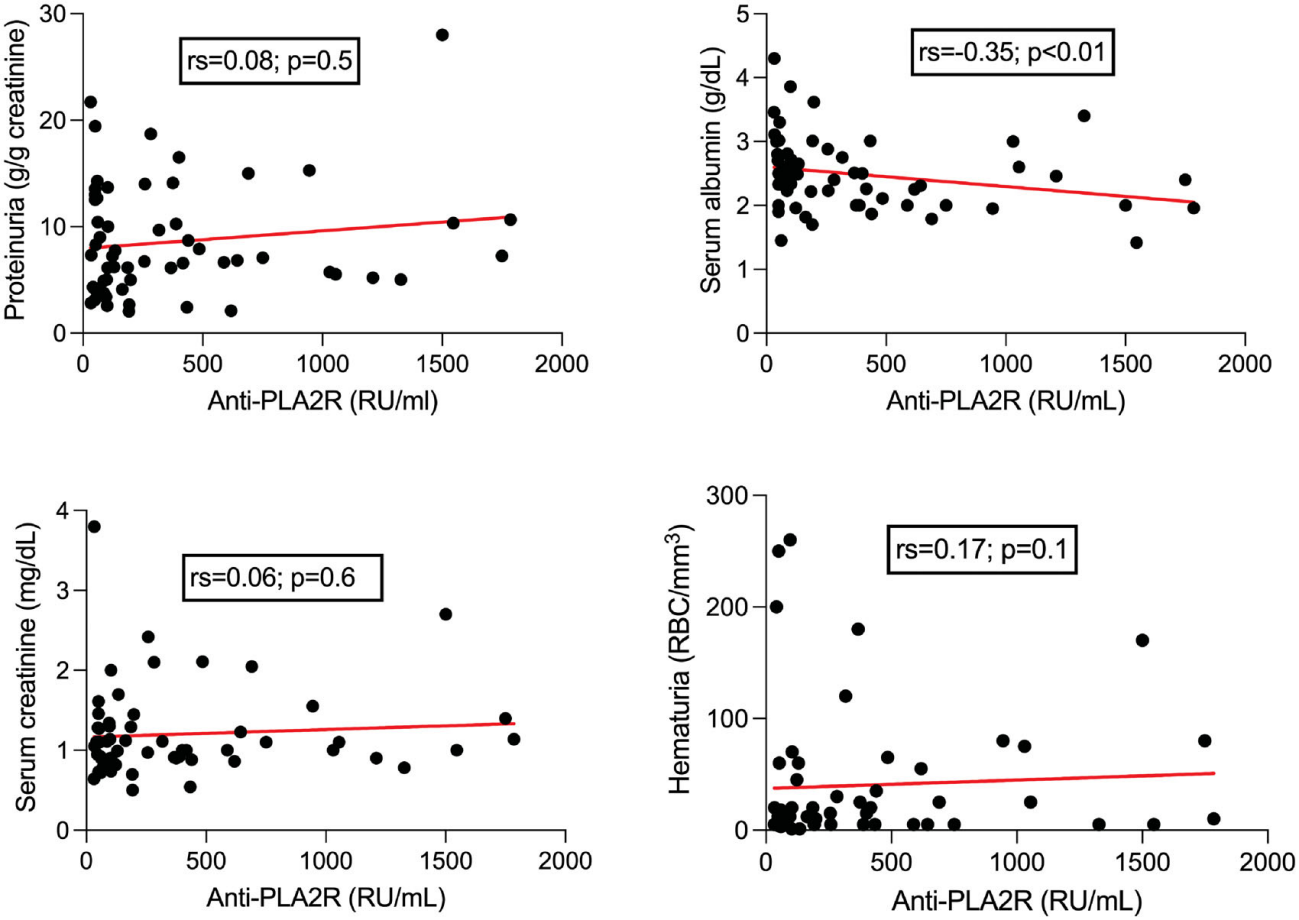
Spearman’s correlation test between anti-PLA2R antibodies titer and proteinuria, serum albumin, serum creatinine, and hematuria at baseline.

At baseline, there was no correlation between the anti-PLA2R antibodies titer and proteinuria, serum creatinine or hematuria in the patients from the outcome cohort. The only significant negative correlation was with serum albumin (Figure 2).

### Anti-PLA2R antibodies titer negativization at three months

Forty (69%) patients from the outcome cohort became anti-PLA2R antibodies negative at 3 months. We found no differences between the two groups at diagnosis regarding age, comorbidities, serum creatinine, serum albumin, and proteinuria. Moreover, there was a similar chronicity pattern at kidney biopsy evaluation, and the MN stages were also comparable. Moreover, patients received in similar proportion immunosuppressive treatment and RAAS blockade (Table 1).

However, patients who experienced the three months anti-PLA2R antibodies negativization had a significantly lower titer of antibodies at baseline (Table 1).

Regarding the outcome, there were no differences in terms of mortality and RRT initiation, but the anti-PLA2R antibodies positive at three months group had the highest proportion of non-responders to treatment (63% versus 22%) (Table 1).

### Response to treatment

Overall, 64% of the patients from the outcome cohort reached a form of remission.

Patients without remission had diabetes mellitus more frequently, more severe nephrotic syndrome (higher proteinuria, lower albuminuria), higher total renal chronicity score and started RRT more often (S1 Figure S2, Table 2).

There was no relationship between the anti-PLA2R antibodies titer at kidney biopsy and remission (S1 Figure S2). However, the proportion of patients who had a negative anti-PLA2R antibodies titer at three months was double in the group who reached remission (Table 2). Moreover, at three months after diagnosis, patients who experienced antibodies negativization had higher serum albumin, but similar proteinuria.

**Table 2.** Baseline patients’ characteristics and anti-PLA2R serology according to remission.

	Remission		*p*
	Yes (*n* = 38)	No (*n* = 21)	
Age (years)	54 [44–68]	58 [48–68]	.4
Male sex (%)	66	76	.5
Hypertension (%)	58	57	.9
Diabetes mellitus (%)	0	24	<.01
Charlson score	0 [0–1]	0 [0–2]	.5
Thrombotic complications (%)	13	10	.6
Anti-PLA2R Ab titer (RU/mL)	131 [62–375]	388 [103–644]	.1
Anti-PLA2R ab negativization at 3 months (%)	82	43	.002
Serum creatinine (mg/dL)	1.0 [0.8–1.2]	1.1 [1.0–1.6]	.09
Serum albumin (g/dL)	2.6 [2.2–3.0]	2.2 [2.0–2.3]	<.01
Proteinuria (g/g)	5.4 [3.7–8.7]	10.3 [7.2–13.6]	<.001
Hematuria (RBC/mm^3^)	12 [5–55]	18 [5–45]	.8
C-reactive protein (mg/L)	1 [1–4]	3 [1–9]	.07
Cholesterol (mg/dL)	336 [272–454]	362 [325–470]	.4
Triglycerides (mg/dL)	204 [173–252]	228 [168–318]	.5
*Kidney biopsy*			
MN stage (%)			.1
I	5	0	
II	61	57	
III	24	43	
IV	10	0	
Total chronicity score	2 [1–4]	4 [1–5]	.05
*Treatment*			
Immunosuppression (%)			.9
Absent	3	5	
Corticotherapy only	8	5	
Cyclophosphamide^a^	87	86	
Cyclosporine^b^	2	4	
RAAS blockade (%)	42	33	.5
*Outcome*			
Death (%)	5	19	.09
RRT initiation (%)	3	19	.03

Ab: antibodies; MN: membranous nephropathy; PLA2R: phospholipase A2 receptor; RAAS: renin–angiotensin–aldosterone system; RBC: red blood cell; RRT: renal replacement therapy.

^a^ Cyclophosphamide (cyclical): methylprednisolone 1 g iv 3 consecutive days in months 1, 3, and 5, prednisone 0.5 mg/kg/d in months 1, 3, and 5; CFM 2.5 mg/kg/d in months 2, 4, and 6. Cyclophosphamide (intravenous pulse): CFM at a dose of 600 mg/m^2^ every 4 weeks – for up to 6 months; in conjunction with prednisolone at a dose of 0.75 mg/kg daily (up to 60 mg/day) – with gradual tapering to 0.5 mg/kg/day by 3 months and 0.1 mg/kg/day by 6 months.

^b^ Cyclosporine 3.5 mg/kg/d and prednisone 10 mg/d – 6–12 months.

Interestingly, the patients from the group that had anti-PLA2R antibodies negativization at three months but did not reach remission had more severe nephrotic syndrome and higher chronicity score on kidney biopsy than those who reached remission and had positive antibodies after three months (S1 Table S3).

Median time to cumulative remission was 12.0 (95%CI, 8.2, 15.7) months and the cumulative remission rates were 34%, 54%, 68%, and 73% after 6, 12, 18, and 24 months. Only five patients relapsed during the study period.

**Figure 3.**
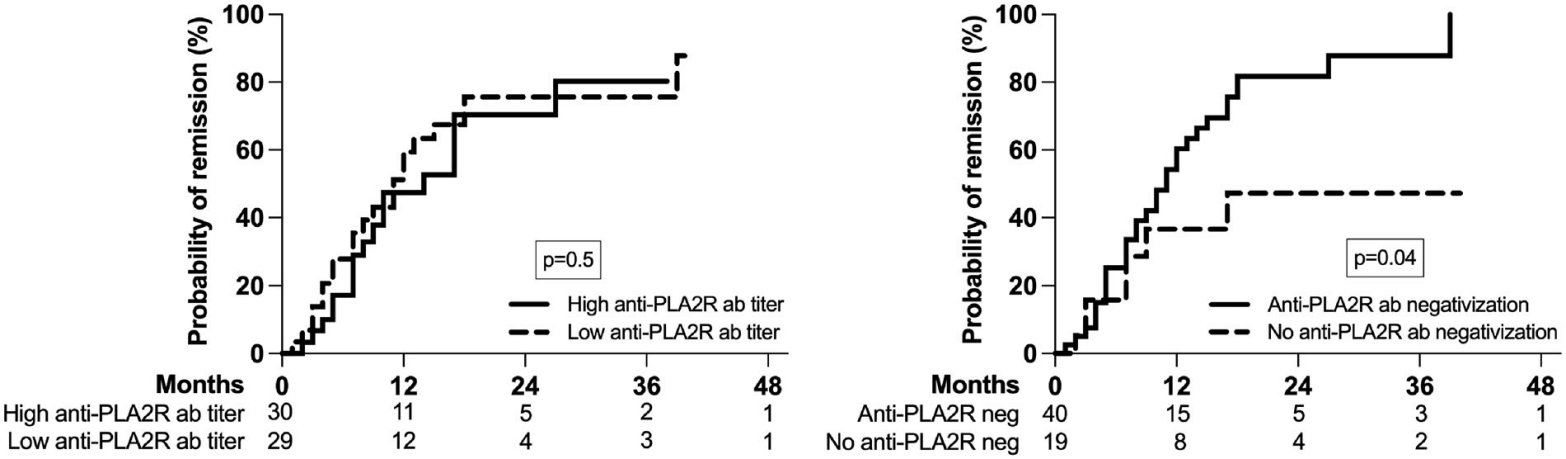
Probability of remission according to anti-PLA2R antibodies titer at baseline (high versus low based on the median value) and the anti-PLA2R antibodies negativization at three months after diagnosis and treatment; the number of patients at risk is shown below the graph.

In univariate time-dependent analysis, there was no difference in the probability of remission according to anti-PLA2R antibodies titer at baseline (high versus low based on the median value), but patients who had anti-PLA2R antibodies negativization at three months had a significantly higher probability of remission (Figure 3). The relationship between baseline characteristics and time to remission is shown in Table 3. We constructed two multivariable Cox regression models: first with the anti-PLA2R antibodies titer at baseline and second with the negativization of the anti-PLA2R antibodies titer in the first three months. While a lower serum albumin was retained as a risk factor for the absence of remission in both models, only the negativization of the anti-PLA2R antibodies at three months – but not their titer at diagnosis – was an independent predictor of remission.

**Table 3.** Univariable and multivariable Cox regression analyses of risk factors for not achieving remission.

	HR (95%CI)	*p*
*Univariable*		
Age (older)	0.99 (0.96, 1.01)	.5
Diabetes mellitus (vs. absent)	2.51 (0.32, 19.60)	.1
Higher PLA2R ab titer	1.00 (0.99, 1.00)	.3
Anti-PLA2R ab negativization (vs. absent)	0.44 (0.19, 1.00)	.05
Higher serum creatinine	0.64 (0.29, 1.42)	.2
Lower serum albumin	2.44 (1.39, 4.30)	.002
Higher proteinuria	0.97 (0.91, 1.03)	.3
Higher total chronicity score	0.96 (0.84, 1.09)	.5
*Multivariable*		
Model 1: anti-PLA2R ab titer at baseline		
Age (older)	0.98 (0.95, 1.01)	.3
Higher serum creatinine	0.74 (0.37, 1.47)	.4
Higher PLA2R ab titer	1.00 (0.99, 1.00)	.5
Higher proteinuria	1.03 (0.98, 1.08)	.2
Lower serum albumin	2.91 (1.54, 5.48)	.001
Model 2: anti-PLA2R ab negativization at 3 months		
Age (older)	0.97 (0.94, 1.00)	.09
Higher serum creatinine	0.83 (0.44, 1.54)	.5
Anti-PLA2R ab negativization at 3 months (vs. absent)	0.40 (0.17, 0.97)	.04
Higher proteinuria	1.04 (0.99, 1.10)	.07
Lower serum albumin	3.02 (1.59, 5.74)	.001

Ab: antibodies; HR: hazard ratio; PLA2R: phospholipase A2 receptor.

### Kidney and patient survival

During the follow up period, six (10%) patients died. Cardiovascular and infectious diseases were the main causes of death. A total of five (9%) patients started RRT during the study period. Only higher serum creatinine at baseline was related to RRT initiation (S1 Figure S3).

**Figure 4.**
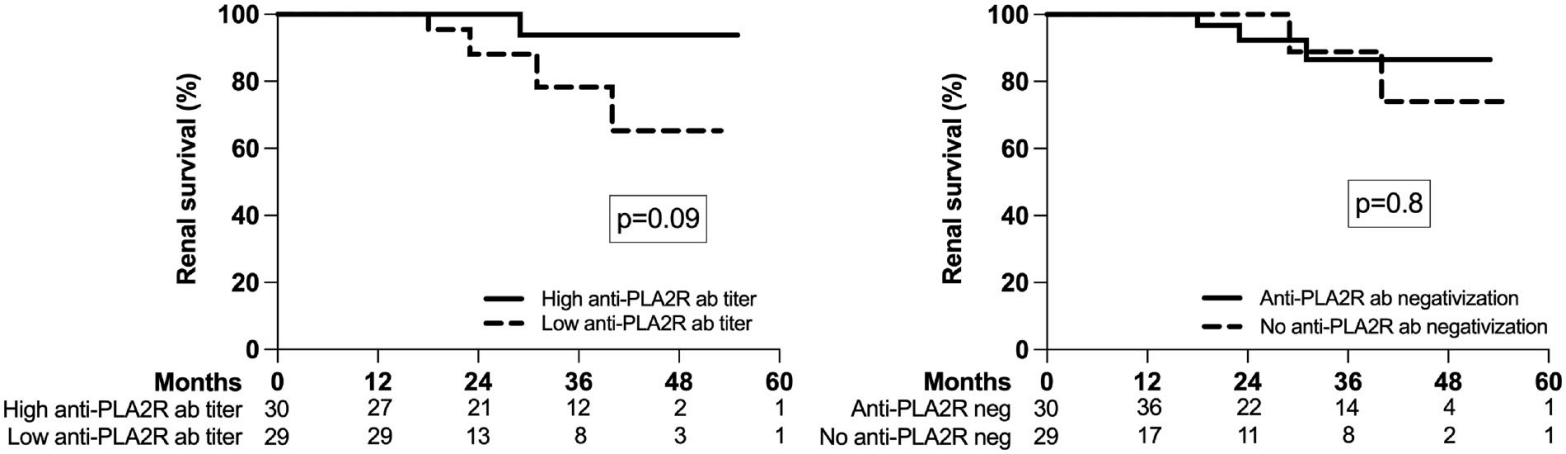
Renal survival according to anti-PLA2R antibodies titer at baseline (high versus low based on the median value) and the anti-PLA2R antibodies negativization at three months after diagnosis and treatment; the number of patients at risk is shown below the graph.

The mean renal survival time was 50.3 (95%CI; 46.5, 54.0) months; renal survival at 12, 24, 36, 48, and 60 months was 98%, 95%, 87%, 81%, and 80%, respectively. We found no difference in renal survival in relation to the anti-PLA2R antibodies titer at baseline or with their three months negativization (Figure 4).

## Discussion

### Prognostic role of anti-PALA2R antibodies titer at diagnosis and at three months

One of the most puzzling issues of primary MN is its wide spectrum of clinical outcomes, in contrast with the paucity of prognostic biomarkers for progression and response to therapy. In this study, we have shown that anti-PLA2R antibodies negativization at three months after diagnosis is a late predictor of remission, while a lower serum albumin at diagnosis is an early predictor of the lack of remission.

### Positive versus negative anti-PALA2R antibodies membranous nephropathy

Membranous nephropathy can be viewed as a group of diseases that share a common histopathologic pattern. Inside this spectrum, primary MN is a kidney-limited autoimmune disease in which circulating antibodies target antigens on podocytes surface [10]. In 70–80% of cases, the target antigen has been identified as the M-type PLA2R 1, in 1–5% the thrombospondin type-1 domain containing 7A and in 5–10% the neural epidermal growth factor-like 1 protein (NELL-1) [2,11,12]. In our study, we assessed the anti-PLA2R antibodies only by ELISA, antibodies anti-THSD7A or NELL1 were not assessed, and kidney biopsies were not stained for the PLA2R and THSD7A antigens. Therefore, the patients in the ‘PLA2R negative group’ were immunologically incompletely characterized. However, the percentage of patients with positive anti-PLA2R antibodies was similar to previous reports (64 vs. 70%) [13], and there were no differences in the clinical phenotype, i.e., proteinuria, serum albumin and kidney function, between groups defined based on the various anti-PLA2R antibodies cutoffs (<2, 2–14, >14 RU/mL) (S1 Figure S1).

In line with other reports, we were unable to find significant differences in baseline clinical phenotype or outcome between anti-PLA2R antibody positive and negative patients in the original cohort [14,15]. In contrast, other studies reported that patients with anti-PLA2R antibodies negative MN had a better prognosis, with higher chances of achieving a clinical remission and a faster decline of proteinuria compared to patients with positive anti-PLA2R antibody [16,17]. However, these studies are limited by the relatively small number of patients with negative anti-PLA2R antibody included (10 and 13, respectively) [16,17].

This suggests that the MN pathogenesis in the anti-PLA2R antibody negative patients might be similar, but other antigens and antibodies could be involved.

In our outcome cohort, the anti-PLA2R antibodies titer at baseline was not correlated with proteinuria, serum creatinine or hematuria. These results are in line with Hoxha et al., who also found no correlation between proteinuria or serum creatinine and total IgG or IgG4 anti-PLA2R antibodies levels at baseline [18], and suggest that PLA2R seropositivity is poorly related to the clinical phenotype.

Remission rates in our study (34% and 54% after 6 and 12 months) were different from those achieved with Ponticelli protocol in two randomized controlled trials with a similar follow up time: 50% at 6 months [19] and 29% within 1 year [20]. However, the remission rates in our outcome cohort were comparable with those reported in patients treated with rituximab [3], cyclosporine [21], and tacrolimus [22], where the remission rates were 60% at 12 months, 75% at 26 weeks, and 58% at 6 months, respectively. These discrepancies could be due to the heterogeneity in the treatment protocol used in our unit: 14% oral cyclophosphamide, 73% pulse cyclophosphamide, 7% corticotherapy only, and 3% cyclosporine.

Although some studies found an association between the remission rate and anti-PLA2R antibodies titer at diagnosis, others found only a weak or no relationship [5,23–29]. This variability most likely reflects the time lag between the immunologic activity and the clinical manifestations. On one hand, as the affinity of antibodies for PLA2R epitope is high, antibodies in low titer are initially rapidly cleared from circulation. Thereafter, the epitopes saturation allows the increase in anti-PLA2R antibodies titer (‘kidney-as-a-sink’ hypothesis) [1]. On the other hand, the local interaction of antibodies with PLA2R antigen, and the activity of the disease, could persist even when serum antibodies titer is low, until the antibodies are cleared from the lesions. Thus, the relationship between the anti-PLA2R antibodies titer and disease activity depends on the moment of serum collection in rapport with the local, glomerular, immunological activity. This is supported by a study which examined serum anti-PLA2R antibodies titer in relation with glomerular expression of PLA2R antigen and immunoglobulin deposition in the kidney. When anti-PLA2R antibodies were present, the clinical phenotype was more severe, probably as glomerular immunoglobulin G4 deposits were more frequent [30].

Decreasing anti-PLA2R antibodies levels appear to be a strong predictor of proteinuria remission [4,18,31]. Radice et al. measured anti-PLA2R antibodies three times in 42 patients with biopsy proven MN and reported that the probability of halving proteinuria increased 6.5 times after negativization of the antibodies [31]. In the GEMRITUX trial, two rituximab infusions were added to the non-immunosuppressive antiproteinuric treatment. Between-group differences in serum albumin preceded those in proteinuria, and a higher percentage increase in serum albumin at months 3 and 6 was associated with an early decrease in anti-PLA2R antibodies titer [32]. Moreover, in a study by Ruggenenti et al., lower anti-PLA2R antibody titer at baseline and antibodies depletion at 6 months after rituximab administration strongly predicted remission: a 50% anti-PLA2R titer reduction preceded an equivalent proteinuria reduction by 10 months [33].

In line, we found that negativization of anti-PLA2R antibodies at three months after diagnosis was an independent predictor of remission in uni- and multivariable analyses and was associated with a 60% increase in chances of remission. Thus, anti-PLA2R antibody negativization at 3 months after diagnosis could be an early marker of remission and this information could guide therapy. Therefore, in patients who have unchanged or increasing serum anti-PLA2R antibodies at 3 months changing the treatment regimen could be the next step, if confirmed in randomized controlled trials.

### Other prognostic factors: hypoalbuminemia and total chronicity score

In multivariable analysis, we also found that a higher serum albumin at the time of MN diagnosis was independently associated with remission in both models. In a study evaluating factors associated with complete remission, low albumin levels at baseline were associated with non-achievement of a complete remission and progression to nephrotic syndrome [34]. Moreover, at three months after diagnosis, patients who experienced antibodies negativization had higher serum albumin. These findings support incorporation of a measure of serum albumin normalization into the definition of partial remission, aiming to significantly improve the precision of clinical endpoints, especially in controlled trials, as suggested by Lee et al. [35].

Similar to Bobart et al., we found that total chronicity score was not correlated with the degree of proteinuria. The patients who did not reach remission had higher chronicity score, probably because of higher proportion of patients with diabetes mellitus, but in the Cox regression analysis the relationship was lost [7]. Moreover, the patients from the group that had anti-PLA2R antibodies negativization at three months but did not reach remission had higher chronicity score. These findings could suggest a possible prognostic role for kidney biopsy in PL2AR-associated MN.

### Relapse rate and kidney survival

The relapse rate of only 9% was rather small in our cohort. Relapses were reported in 25–40% of MN cases, and the relapses frequency in MN is time-dependent and the reported relapses cumulative rate was 36% and 44% at 12 and 24 months after the primary remission [36]. Accordingly, the low rate of relapse in our cohort may be explained by the short median follow-up time (only 21 months).

Overall kidney survival in MN was 86% at five years and decreased to 65% at 10 years and alkylating agents, not corticoids, improved kidney survival in a meta-analysis by Hogan et al. [37]. Also, du Buf-Vereijken et al. pooled data from 10 studies published in over 25 years, excluding those with short follow-up, and found ESKD in 10–30% of patients with MN at 10 years [38]. Moreover, Jha et al. found a better 10-year renal survival in MN nephrotic patients treated with corticoids and cyclophosphamide as compared to supportive care: 89 versus 65% [20]. In our cohort, we observed a similar 80% kidney survival rate at five years, but neither the baseline titer of anti-PLA2R antibodies, nor its negativization at three months predicted RRT initiation, which could also reflect a variable time lag between immunological activity and kidney lesion. Nevertheless, our kidney survival analysis could be hampered by the small population and the relatively short follow up time.

### Study limitations

We recognize several limitations of our study. Data were retrospectively collected and were, therefore, dependent on the accuracy and completeness of the electronic databases. Moreover, our outcome cohort is rather small and due to the referral-based nature of our population it may not be representative for all adult patients with MN. Also, nonuniformity use of immunosuppression hampered comparison of the effectiveness of the drugs. Staining for PLA2R antigen was not available, therefore PL2AR-associated MN may have been missed in those cases with negative serology.

## Conclusions

In conclusion, anti-PLA2R antibodies titer negativization at three months may predict remission in primary MN. Therefore, it is important to monitor – at least at three months – the anti-PLA2R antibodies titer during the treatment. However, the severity of hypoalbuminemia at diagnosis also predicts the absence of remission.

## Supplementary Material

Supplemental Material

## Data Availability

The datasets used and/or analyzed during the current study are available from the corresponding author on reasonable request.
