# Supplementary material for: Negative anti-phospholipase A2 receptor antibody status at three months predicts remission in primary membranous nephropathy
Source: Ren Fail. 2022 Feb 16;44(1):258–68. doi: 10.1080/0886022X.2022.2033265 (PMC8863379; doi:10.1080/0886022X.2022.2033265)
Supplement: Supplemental Material [file IRNF_A_2033265_SM3632.pdf]

Supporting Information

S1 Table 1. Definitions of histologic variables

	**Definition**
Light microscopy	
Glomerulosclerosis (GS)	Percentage of glomeruli with global sclerosis and ischemic glomeruli: 0, <10%; 1, 10-25%; 2, 26-50%; 3, >50%
Interstitial fibrosis (IF)	Percentage of renal cortex involved: 0, <10%; 1, 10-25%; 2, 26-50%; 3, >50%
Tubular atrophy (TA)	Percentage of renal cortex involved: 0, <10%; 1, 10-25%; 2, 26-50%; 3, >50%
Arteriosclerosis	Extent of thickening of the intima: 0, intimal thickening < thickness of media; 1, intimal thickening ≥ thickness of media
Total renal chronicity score	The scores of GS, TA, IF and arteriosclerosis are added to grade the overall severity into minimal (0–1 total score), mild (2–4 total score), moderate (5–7 total score), and severe (≥ 8 total score)
Membranous nephropathy	
Stage I	Immune-complex-type electron-dense deposits in the subepithelial spece between the basement membrane and the podocyte
Stage II	Projections of basement membrane material around the subepithelial deposits
Stage III	New basement membrane material surrounds the deposits
Stage IV	Loss of electron density of the deposits, which results in irregular electron-lucent zones

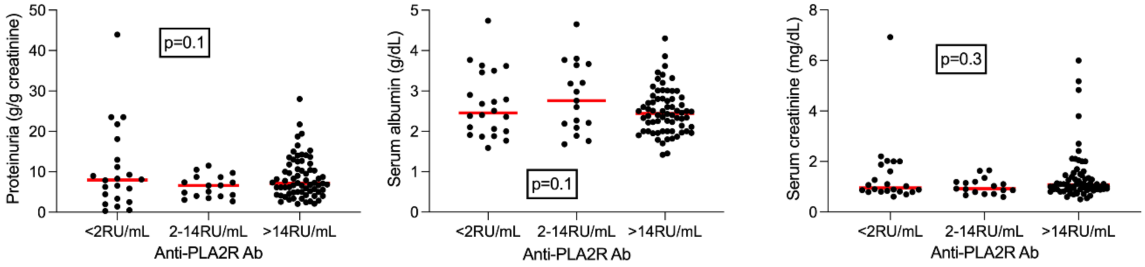

S1 Figure S1. Comparisons between patient groups defined based on various anti-PLA2R antibodies titer cut-offs (<2, 2-14, >14RU/mL)

S1 Table S2. Original cohort patients' characteristics at baseline according to anti-PLA2R antibodies serology

	**All** (N=107)	**PLA2R Ab**		**p**
		**Positive** (n=68)	**Negative** (n=39)	
Age (years)	56 [42-68]	54 [44-68]	58 [41-67]	0.8
Male sex (%)	65	69	62	0.5
Hypertension (%)	60	62	51	0.2
Diabetes mellitus (%)	10	9	13	0.5
Charlson score	1 [0-2]	3 [1-5]	2 [0-4]	0.3
Thrombotic complications (%)	12	12	13	0.8
Serum creatinine (mg/dL)	1.0 [0.8-1.4]	1.0 [0.9-1.4]	0.9 [0.8-1.3]	0.2
Serum albumin (g/dL)	2.4 [2.0-3.0]	2.4 [2.0-2.8]	2.6 [2.0-3.5]	0.2
Proteinuria (g/g)	7.2 [4.3-10.6]	7.2 [4.9-11.9]	6.6 [3.9-9.7]	0.3
Hematuria (RBC/mm^3)	12 [5-30]	12 [5-35]	11 [5-30]	0.8
C-reactive protein (mg/L)	2 [1-6]	0 [0-2]	1 [0-2]	0.3
Cholesterol (mg/dL)	336 [256-420]	345 [275-451]	313 [232-412]	0.08
Triglycerides (mg/dL)	203 [155-265]	224 [166-282]	180 [129-247]	0.03
**Kidney biopsy**				
MN stage (%)				0.01
I	9	3	21	
II	49	54	39	
III	32	31	33	
IV	10	12	7	
Total chronicity score	2 [1-5]	3 [1-5]	2 [0-4]	0.1
**Treatment**				
Immunosuppression (%)				<0.01
Absent	10	5	21	
Corticotherapy only	10	10	10	
Cyclophosphamide	73	82	56	
Cyclosporine	7	3	13	
RAAS blockade (%)	36	38	33	0.6
**Outcome**				
Death (%)	8	9	5	0.4
RRT initiation (%)	7	9	3	0.2
Ab, antibodies; MN, membranous nephropathy; PLA2R, phospholipase A2 receptor; RAAS, renin-angiotensin-aldosterone system; RBC, red blood cells; RRT, renal replacement therapy				

S1 Table S3. Baseline characteristics of patients anti-PLA2R antibodies negativization at three months but without remission versus patients without anti-PLA2R antibodies negativization but with remission

**Anti-PLA2R Ab negativization at 3 months**	**Present**	**Absent**	**p**
**Remission**	**Absent**	**Present**	
Number of patients	8	7	
Age (years)	64 [54-72]	54 [45-71]	0.2
Male sex (%)	63	71	0.7
Hypertension (%)	63	71	0.7
Diabetes mellitus (%)	25	0	0.1
Charlson score	1 [0-3]	1 [0-1]	0.8
Thrombotic complications (%)	13	0	0.3
Anti-PLA2R Ab (RU/mL)	103.5 [50.0-261.3]	367.0 [100.3-1031.0]	0.1
Serum creatinine (mg/dL)	1.0 [0.8-1.3]	0.9 [0.9-1.0]	0.5
Serum albumin (g/dL)	2.1 [1.9-2.4]	2.5 [2.3-3.0]	0.02
Proteinuria (g/g)	13.0 [8.6-15]	5.2 [2.5-6.1]	<0.01
Hematuria (RBC/mm^3)	18 [8-58]	55 [15-75]	0.3
C-reactive protein (mg/L)	4 [1-8]	1 [1-11]	0.5
Cholesterol (mg/dL)	360 [290-455]	382 [281-533]	0.8
Triglycerides (mg/dL)	193 [174-233]	291 [168-355]	0.2
Total chronicity score	5 [4-5]	1 [0-3]	0.02
Immunosuppression (%)			0.5
Absent	13	0	
Corticotherapy only	13	0	
Cyclophosphamide	61	86	
Cyclosporine	13	14	
RAAS blockade (%)	25	43	0.4
Death (%)	25	0	0.1
RRT initiation (%)	25	0	0.1
Ab, antibodies; MN, membranous nephropathy; PLA2R, phospholipase A2 receptor; RAAS, renin-angiotensin-aldosterone system; RBC, red blood cells; RRT, renal replacement therapy			

S1 Table S4. Serum creatinine, serum albumin, proteinuria, and hematuria at three months after diagnosis according to anti-PLA2R antibodies negativization status

	**Three months anti-PLA2R ab negativization**		**p**
	**Yes (n=40)**	**No (n=19)**	
Serum creatinine (mg/dL)	1.0 [0.8-1.2]	1.1 [0.8-1.4]	0.4
Serum albumin (g/dL)	3.0 [2.7-3.5]	2.8 [2.2-3.0]	0.02
Proteinuria (g/g)	4.5 [2.2-7.6]	5.6 [2.3-11.3]	0.3
Hematuria (RBC/mm^3)	5 [5-12]	6 [5-15]	0.5
Ab, antibodies; PLA2R, phospholipase A2 receptor; RBC, red blood cells			

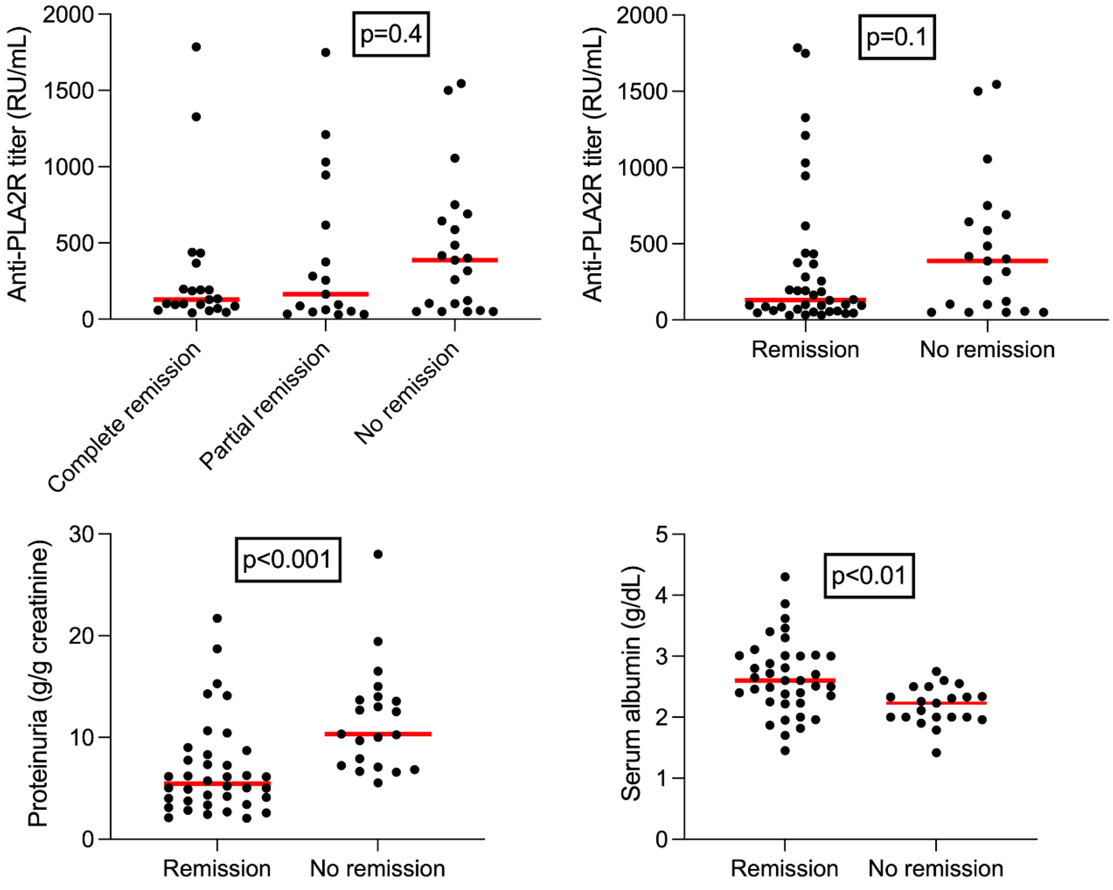

S1 Figure S2. Comparisons between baseline parameters according to remission status

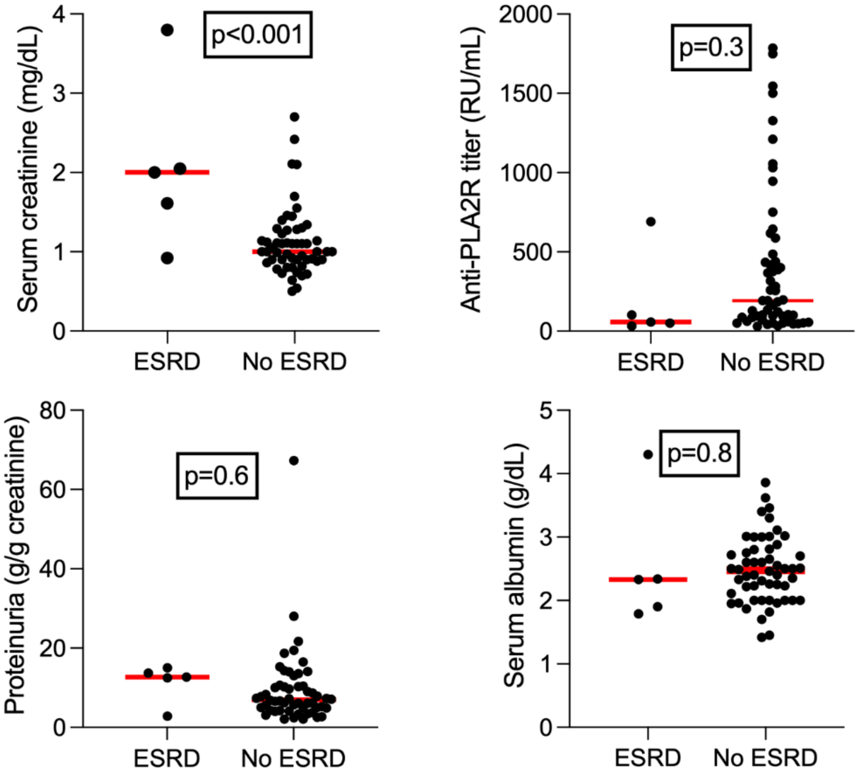

S1 Figure S3. Comparison between patients who started renal replacement therapy in the study period (ESRD, end stage renal disease)
